# Unmasking vestibular schwannoma: A series of unusual cases

**DOI:** 10.1016/j.ijscr.2025.111291

**Published:** 2025-04-12

**Authors:** Nataly Haider, Qais Radi, Solaf Ahmad, Samer Mohsen

**Affiliations:** a Faculty of Medicine, Damascus University, Damascus, Syria; b Department of Otolaryngology, Faculty of Medicine, Damascus University, Damascus, Syria.; c Department of Audiology, Faculty of Health Sciences, Damascus University, Damascus, Syria

**Keywords:** Acoustic neuroma, Retrocochlear lesion, Case report

## Abstract

**Introduction:**

Vestibular schwannoma (VS) is a benign, slow-growing tumor that accounts for 80–90 % of cerebellopontine angle (CPA) tumors. While classic symptoms like hearing loss, tinnitus, and vertigo are well-recognized, atypical presentations can delay diagnosis.

**Case Presentation:**

We present four unusual cases of VS. The first patient, a 33-year-old woman, initially presented with transient tinnitus and later developed peripheral vertigo. The second case involved a 52-year-old man with sudden hearing loss, vertigo, and aural fullness. The third case was a 17-year-old female with unilateral auditory neuropathy. The fourth patient, a 39-year-old woman, presented with intermittent tinnitus, balance disturbances, and facial stiffness.

**Discussion:**

Detailed history, physical examination, and audiological evaluation are crucial for identifying patients at risk for vestibular schwannoma, especially with atypical audiological findings, to avoid misdiagnosis.

**Conclusion:**

This case series emphasizes the significance of considering VS as a differential diagnosis for atypical symptoms. Early recognition and proper practice can lead to optimal outcomes for patients and contribute valuable insights to the scientific field by broadening the understanding of the diverse spectrum of VS symptoms.

## Highlights

• Unusual audiological profiles stress the need for vigilance to avoid delays in diagnosis and treatment.
• Small or atypically located tumors can hinder early detection, complicating diagnosis and management.
• Prompt diagnosis using MRI is essential for the best possible management and prognosis.
• An absent ABR with a preserved CM could result in the misdiagnosis of auditory neuropathy.

## 1 Introduction

Vestibular schwannoma (VS), also known as acoustic neuroma, is a benign, slow-growing tumor arising from Schwann cells of the vestibulocochlear nerve [[1], [2], [3]]. While typically originating from the inferior vestibular nerve, it can occasionally arise from the superior vestibular or cochlear nerve branches [4].

As one of the most common tumors of the posterior fossa, VS accounts for approximately 6–9 % of all brain tumors and over 80–90 % of cerebellopontine angle (CPA) tumors [2,5] The incidence of VS has been increasing, reaching 1.29 per 100,000 populations in recent years [6].

The classic presentation of VS involves a triad of unilateral sensorineural hearing loss, tinnitus, and balance disturbances [7,8]. However, the insidious onset of symptoms often delays diagnosis. Hearing loss is the most common presenting symptom, prompting further investigation. Other symptoms may include imbalance, facial numbness, headache, visual changes, otalgia, facial weakness, nausea, cranial nerve palsy, facial nerve palsy, varied morphologies of PAI, compression of nearby cranial nerves, etc. In many cases, symptoms develop slowly and might be mistaken for other conditions so that diagnosis can be delayed [9,10]. Large tumors can cause more severe symptoms such as facial paresthesia, vertigo, and headache due to compression of the brainstem and hydrocephalus [7,11,12].

Atypical presentations of VS can pose diagnostic challenges, as they may mimic other neurological or otological disorders. These unusual presentations include sudden sensorineural hearing loss, facial numbness, headaches, and cerebellar signs like ataxia or uncoordinated movements [13]. Trigeminal neuralgia, diplopia, and other cranial nerve deficits can also occur, depending on the tumor's location and size [14].

The gold standard for diagnosing VS remains gadolinium-enhanced magnetic resonance imaging)MRI(of the internal auditory canal and cerebellopontine angle [15]. Treatment options include observation, surgical resection, and radiation therapy [16]. Preoperative and intraoperative considerations play a pivotal role in ensuring successful tumor resection. Preoperative CT and MRI imaging of the porus acusticus internus)PAI(are essential for precise tumor localization and surgical planning. Intraoperatively, the PAI serves as a critical anatomical reference for safe tumor removal, and a thorough understanding of its anatomical variations can significantly reduce the risk of nerve injury [10].

Moreover, ethnic variations in the anatomical structure of the internal auditory canal (IAC), including differences in size and shape, may influence both surgical planning and radiological assessments. Such anatomical discrepancies necessitate careful preoperative evaluation to optimize surgical outcomes [10].

This case series highlights four unusual presentations of VS, collected retrospectively from a private clinic, emphasizing the importance of considering this diagnosis in patients with atypical symptoms. Early diagnosis and appropriate management are crucial for optimal patient outcomes.

This work has been reported in line with the PROCESS criteria [17].

## 2 Case presentation 1

**Fig. 1:**
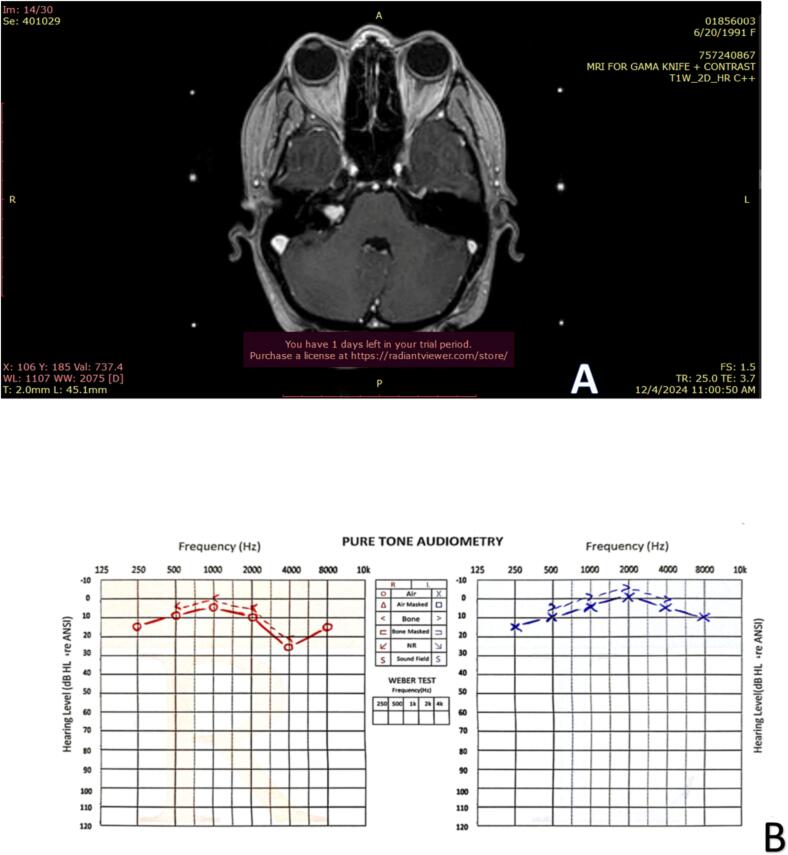
Case 1. **A:** Magnetic resonance imaging (MRI) revealed a 1.2 × 0.6 × 0.6 cm tumor in the right cerebellopontine angle (CPA). **B:** Pure Tone Audiometry revealed a mild high-frequency hearing loss in the right ear (10, 15 dB on 6, 8 K Hz respectively).

A 33-year-old woman presented with a three-month history of episodic, high-pitched tinnitus in her right ear lasting for approximately five minutes. Initial evaluation suggested Eustachian tube dysfunction due to seasonal allergy, and appropriate treatment was initiated. However, subsequent evaluation revealed absent acoustic reflexes in the right ear, with normal impedance audiometry and speech discrimination. Pure tone audiometry demonstrated mild high-frequency hearing loss in the affected ear (Fig. 1. B). Magnetic resonance imaging (MRI) confirmed the presence of vestibular schwannoma in the right cerebellopontine angle (CPA) (Fig. 1. A).

The patient was scheduled for Gamma Knife radiosurgery. Three weeks later, she experienced acute sensorineural hearing loss, with a 30 dB loss in mid-frequencies and a 50 dB loss in high frequencies. Corticosteroid therapy was initiated, and a repeat MRI showed no change in tumor size or evidence of hemorrhage. Following treatment, her hearing thresholds returned to pre-treatment levels.

## 3 Case presentation 2

A 52-year-old man presented with a sudden onset of left-sided sensorineural hearing loss, vertigo, and aural fullness following an acute upper respiratory tract infection. Subsequently, he developed episodic, positional vertigo. His medical history included hypertension and type 1 diabetes mellitus.

**Fig. 2:**
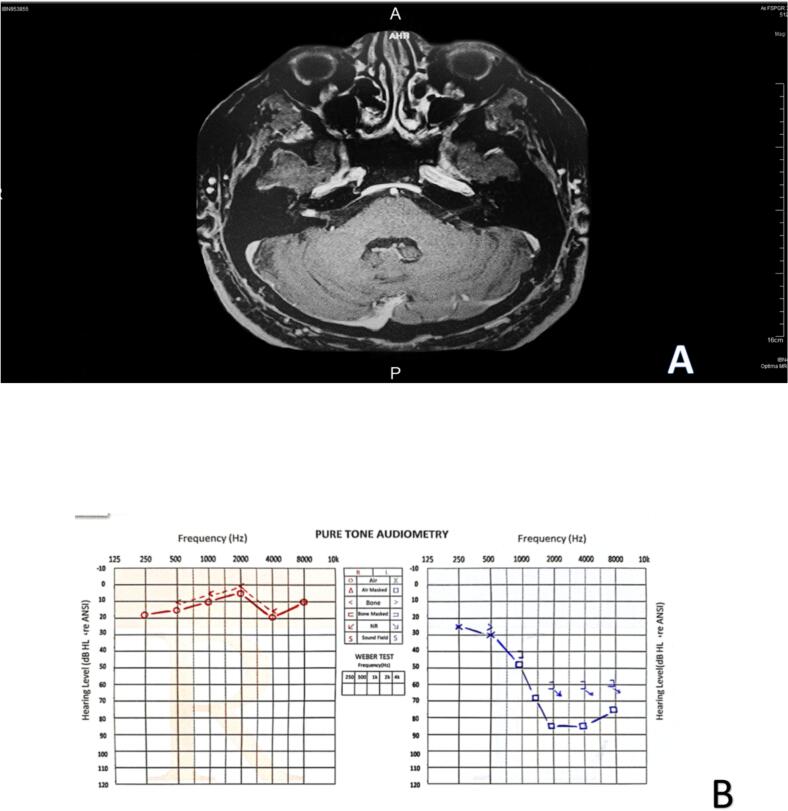
Case 2. **A:** The MRI revealed a small enhancing mass lesion measuring 5 mm, seen within the distal part of the right internal auditory canal. **B:** Pure tone audiometry demonstrated severe sensorineural hearing loss in the left ear at frequencies above 500 Hz and normal thresholds in the right ear.

Otoscopic examination was unremarkable. Vestibular testing revealed bilateral vestibular weakness, with more severe impairment on the left side (Table 1). Pure tone audiometry demonstrated a severe sensorineural hearing loss in the left ear at frequencies above 500 Hz (Fig. 2. B).

**Table 1:** the auditory tests we did to the patient.

Name of the test	Result
Gaze test	Positive
Smooth pursuit test	right beat nystagmus
Head impulse test HIT	right beat nystagmus
Romberg test	Positive
Fukuda Step Test	Left
Dix hall pike test	Positive

Magnetic resonance imaging (MRI) revealed a small (3 mm) possible intracanalicular acoustic neuroma on the right side, with normal appearance of both cerebellopontine angles and internal acoustic meatus. Additionally, an incidental small (13 mm) arachnoid cyst was noted, slightly indenting the inferior left cerebellar hemisphere.

A follow-up MRI after one year showed a small enhancing mass lesion (5 mm) in the distal part of the right internal auditory canal, consistent with a growing acoustic neuroma (Fig. 2. A).

## 4 Case presentation 3

**Fig. 3:**
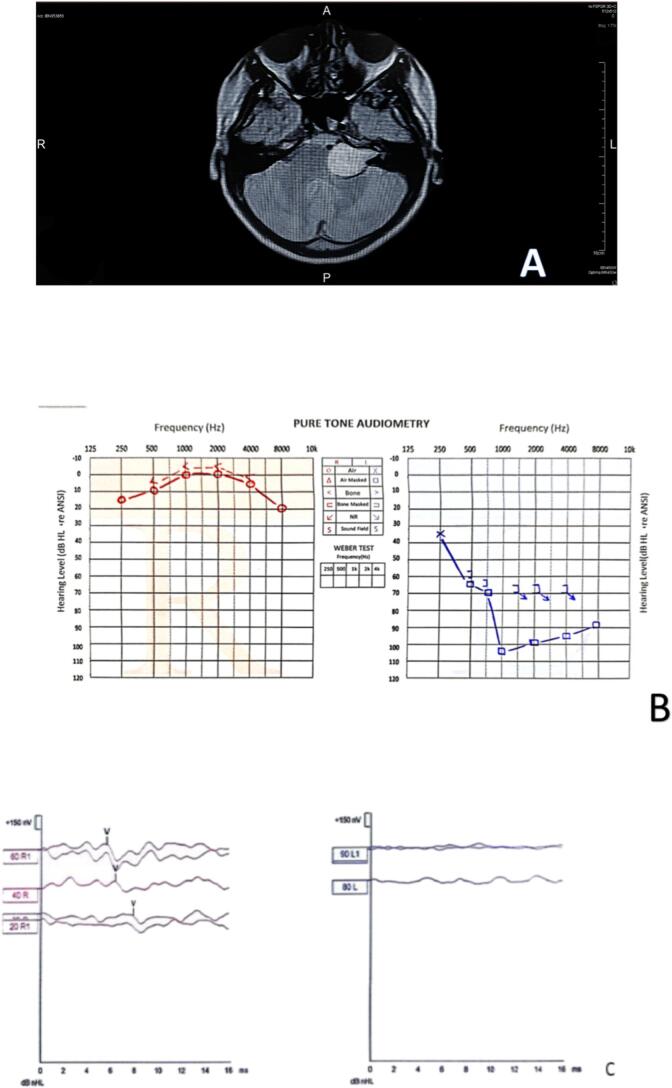
Case 3. **A:** The MRI revealed a 3 cm tumor arising from the left eighth cranial nerve in the cerebellopontine angle. **B:** Pure tone Audiometry revealed a sensory/neural hearing loss in the left ear on 500 K.HZ. **C:** Brainstem auditory evoked responses (ABR) were absent until 90 dB in the left ear and appeared at 20 dB in the right ear.

A 17-year-old healthy female presented to an otolaryngology clinic with a six-month history of left-sided hearing loss. Audiological evaluation revealed a sensory/neural hearing loss in the left ear (Fig. 3. B), poor speech discrimination, absent acoustic reflexes, and absent brainstem auditory evoked responses (ABR) (Fig. 3. C) with preserved cochlear microphonic (CM) (Table 2). These findings suggested a retrocochlear lesion, prompting a recommendation for magnetic resonance imaging (MRI).

**Table 2:** characteristics of case 3.

Name of the test	Result
pure tone audiometry	Sensorineural hearing loss in the left ear
speech discrimination score	Low
acoustic reflex	Absent
ABR	Absent in the left side
Cochlear microphonics	Positive
OAE	Pass

However, the patient initially declined the MRI and sought a second opinion, where she was diagnosed with unilateral acquired auditory neuropathy. She was advised to try a hearing aid and undergo an auditory training program.

After two months without improvement, the patient returned for reevaluation. Given the persistent hearing loss and the initial audiological findings, an MRI was strongly recommended and subsequently performed. The MRI revealed a 3 cm tumor arising from the left eighth cranial nerve in the cerebellopontine angle (Fig. 3. A). The tumor was successfully surgically removed.

## 5 Case presentation 4

A 39-year-old woman presented with a four-month history of intermittent tinnitus that initially affected the left ear and later progressed to bilateral involvement. She also complained of balance disturbances, a tendency to fall, and facial stiffness.

**Fig. 4:**
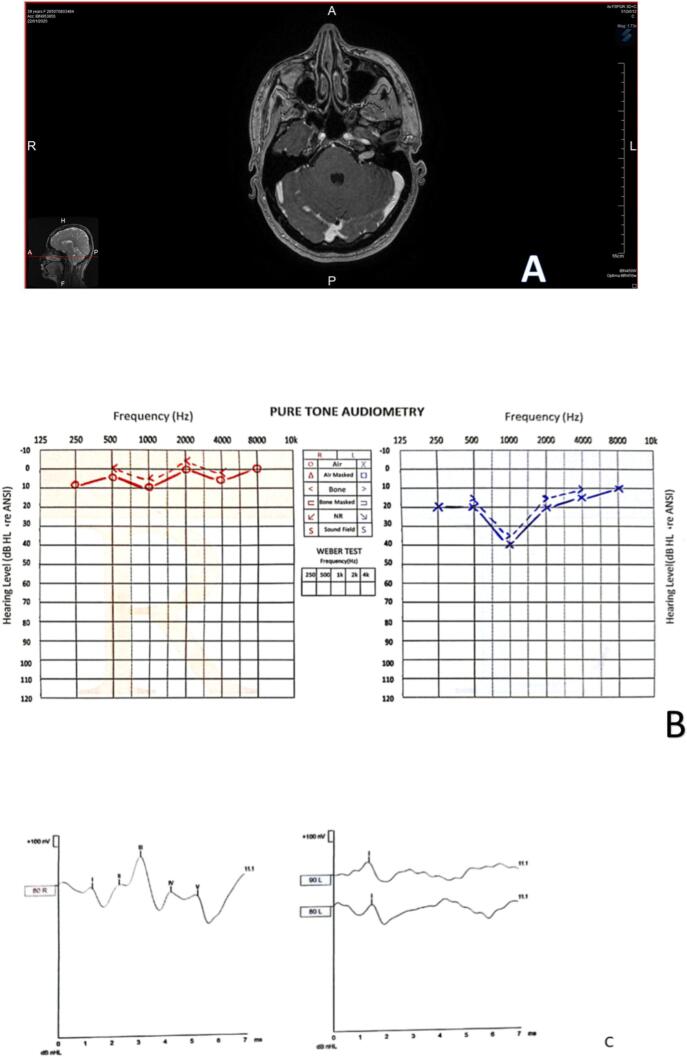
Case 4. **A:** The MRI revealed a (6 × 6 × 12) mm tumor within the left internal auditory canal. **B:** Pure-tone audiometry showed a mild hearing loss notched at 1000 Hz in the left ear. **C:** Auditory brainstem responses (ABR) were absent until 90 dB on the left ear and normal on the right ear.

Audiological evaluation revealed absent acoustic reflexes in both ears. Pure-tone audiometry showed a mild hearing loss notched at 1000 Hz in the left ear (Fig. 4. B). Auditory brainstem response (ABR) testing was absent on the left side (Fig. 4. C), and speech discrimination scores were significantly reduced.

Given the clinical presentation and audiological findings, a magnetic resonance imaging (MRI) of the brain was performed. The MRI revealed a 1 cm tumor within the internal auditory canal, consistent with an acoustic neuroma (Fig. 4. A). The small size of the tumor could explain the severity of symptoms due to its location and impact on the auditory and vestibular nerve functions.

## 6 Discussion

This case series highlights four unusual presentations of vestibular schwannoma, a benign tumor affecting the eighth cranial nerve. While classic presentations often involve hearing loss, tinnitus, and balance disturbances [[7], [8], [9]]. These cases demonstrate the diverse and atypical clinical manifestations of this condition.

The atypical presentations in these cases underscore the need for a high index of suspicion and thorough diagnostic evaluation to avoid delays in diagnosis and mismanagement. In a recent study, it is recommended to study asymmetric hearing loss with MRI when there is asymmetry of at least 20 dB in the 4000 Hz frequency [18].The variability in symptomatology emphasizes the importance of personalized treatment approaches, ranging from conservative observation to aggressive surgical intervention, depending on factors such as tumor size, location, and patient-specific characteristics [16,19,20].

Early diagnosis through advanced imaging techniques, such as MRI, is crucial for optimal management and prognosis. In some cases, the tumor may be small or located in an atypical position, making early detection challenging [11,21].

Therefore, a detailed history, physical examination, and audiological evaluation are essential to identify patients at risk for VS. It is important to note that atypical audiological findings, such as absent ABR with preserved CM, may lead to a misdiagnosis of auditory neuropathy. In such cases, a high index of suspicion for a retrocochlear lesion, such as a vestibular schwannoma, should be maintained. Two of our cases exemplify this scenario: one patient had a 3 cm tumor in the CPA, while another had a 1 cm tumor in the auditory canal. Despite the absence of ABR and the lack of typical VS-related ABR findings, these cases highlight the importance of considering VS in patients with such atypical audiological profiles.

While surgical intervention is often the standard treatment for VS, the decision-making process must consider the potential risks and benefits. In cases where the tumor is small and slow-growing, observation may be a reasonable approach. However, for larger tumors or those with rapid growth, surgical resection or stereotactic radiosurgery may be necessary [22]. As illustrated in the first case, even brief episodes of tinnitus can be a red flag for potential VS, especially when accompanied by other atypical symptoms. For instance, there is a case of vestibular schwannoma was reported with continuous numbness over the right cheek and chin, accompanied by mild tinnitus on the right side [21]. Timely diagnosis and intervention are crucial to optimize patient outcomes.

In conclusion, These cases emphasize that we should not ignore and keep in mind the VS as a differential diagnosis of patients with atypical symptoms like episodic or intermittent unilateral tinnitus, sudden onset of sensorineural hearing loss, and unilateral acquired auditory neuropathy This will help the medical members to Early diagnosis and intervention to reach optimal patient outcomes and contribute novel insight for the scientific field by expanding the understanding of the broad spectrum of VS symptoms. A multidisciplinary approach involving otolaryngologists, neurologists, and neurosurgeons is essential to optimize patient outcomes.

Future research should employ advanced methodologies to strengthen the evidence base, explore the frequency of these symptoms, and investigate the potential presence of additional atypical symptoms.

## Consent

Written informed consent was obtained from the patient's parents/legal guardian for publication and any accompanying images. A copy of the written consent is available for review by the Editor-in-Chief of this journal on request.

## Ethical approval

The cases were taken from a private clinic in Damascus, Damascus University and the ministry of health do not require ethical approval for case reports or case series.

## Guarantor

Samer Mohsen.

## Funding

The study was not funded.

## Authors contribution

**Nataly Haider, Qais Radi, and Solaf Ahmad** have contributed equally to preparing this manuscript;

**Samer Mohsen.** Collected data wrote the manuscript, and reviewed the manuscript.

## Registration of research studies

The study is not registered.

## Conflicts of interest

The authors declare no conflict of interest.

## Data Availability

All data related to these four cases are available depending on the consent and approval given by the patient.

## References

[1] Singh K., Singh M.P., Cl Thukral, Rao K., Singh K., Singh A. (2015). Role of magnetic resonance imaging in evaluation of cerebellopontine angle Schwannomas. Indian J. Otolaryngol. Head Neck Surg..

[2] Lin D., Hegarty J.L., Fischbein N.J., Jackler R.K. (2005). The prevalence of “incidental” acoustic neuroma. Arch Otolaryngol Neck Surg..

[3] Schwannomas Vestibular (2011). Lessons for the neurosurgeon part II molecular biology and histology. Contemp Neurosurg..

[4] Khrais T., Romano G., Sanna M. (2008). Nerve origin of vestibular schwannoma: a prospective study. J. Laryngol. Otol..

[5] Weiss N.M., Großmann W., Schraven S.P., Oberhoffner T., Mlynski R. (2021). Neuromonitoring des N. Cochlearis bei der Resektion des Vestibularisschwannoms mit simultaner Cochleaimplantation. HNO.

[6] Marinelli J.P., Lohse C.M., Grossardt B.R., Lane J.I., Carlson M.L. (2020). Rising incidence of sporadic vestibular schwannoma: true biological shift versus simply greater detection. Otol. Neurotol..

[7] Gupta V.K., Thakker A., Gupta K.K. (2020). Vestibular schwannoma: what we know and where we are heading. Head Neck Pathol..

[8] Connor S.E.J. (2021). Imaging of the vestibular schwannoma. Neuroimaging Clin. N. Am..

[9] Moffat D.A., Baguley D.M., Von Blumenthal H., Irving R.M., Hardy D.G. (1994). Sudden deafness in vestibular schwannoma. J. Laryngol. Otol..

[10] Sekerci R., Ogut E., Keles-Celik N. (2021). The influences of Porus acusticus internus on ethnicity and importance in preoperative and intraoperative approaches. Surg. Radiol. Anat..

[11] Kentala E., Pyykkö I. (2001). Clinical picture of vestibular schwannoma. Auris Nasus Larynx.

[12] Levy R.A., Arts H.A. (1996). Predicting neuroradiologic outcome in patients referred for audiovestibular dysfunction. AJNR Am. J. Neuroradiol..

[13] Irfan S, Kadam AD, Ravichandran U. An atypical presentation of acoustic neuroma with facial paresthesia: a case report. *Cureus*. Published online March 22, 2024. doi:10.7759/cureus.56745. PMC11033451 38650777

[14] Carlson M.L., Link M.J. (2021). Vestibular Schwannomas. Ingelfinger JR, ed. N. Engl. J. Med..

[15] Dang L., Tu N.C., yau, Chan EY. (2020). Current imaging tools for vestibular schwannoma. Curr. Opin. Otolaryngol. Head Neck Surg..

[16] Halliday J., Rutherford S.A., McCabe M.G., Evans D.G. (2018). An update on the diagnosis and treatment of vestibular schwannoma. Expert. Rev. Neurother..

[17] Mathew G, Sohrabi C, Franchi T, et al. Preferred reporting of case series in surgery (PROCESS) 2023 guidelines. *Int. J. Surg.* Published online November 21, 2023. doi:10.1097/JS9.0000000000000940. PMC10720832 37988417

[18] Celis-Aguilar E, Obeso-Pereda A, Castro-Bórquez KM, Dehesa-Lopez E, Vega-Alarcon A, Coutinho-De Toledo H. Multiple audiometric analysis in the screening of vestibular schwannoma. *Cureus*. Published online January 22, 2022. doi:10.7759/cureus.21492. PMC8783613 35103228

[19] Carlson M.L., Lees K.A., Patel N.S. (2016). The clinical behavior of asymptomatic incidental vestibular Schwannomas is similar to that of symptomatic tumors. Otol. Neurotol..

[20] Cueva R.A. (2010). Clinical thresholds for when to test for Retrocochlear lesions: pro. Arch Otolaryngol Neck Surg..

[21] Lim C.C., Misron K., Liew Y.T., Wong E.H.C. (2019). Acoustic neuroma with orofacial paresthesia: description of an atypical presentation. BMJ Case Rep..

[22] Ruiz-García C., Lassaletta L., López-Larrubia P., Varela-Nieto I., Murillo-Cuesta S. (2024). Tumors of the nervous system and hearing loss: beyond vestibular schwannomas. Hear. Res..

